# Comparative phylogenomic analysis provides insights into TCP gene functions in *Sorghum*

**DOI:** 10.1038/srep38488

**Published:** 2016-12-05

**Authors:** Aleena Francis, Namrata Dhaka, Mohit Bakshi, Ki-Hong Jung, Manoj K. Sharma, Rita Sharma

**Affiliations:** 1 School of Computational and Integrative Sciences, Jawaharlal Nehru University, New Mehrauli Road, New Delhi, 110067, India; 2 Graduate School of Biotechnology & Crop Biotech Institute, Kyung Hee University, Yongin, 17104, Republic of Korea; 3 School of Biotechnology, Jawaharlal Nehru University, New Mehrauli Road, New Delhi, 110067, India

## Abstract

*Sorghum* is a highly efficient C4 crop with potential to mitigate challenges associated with food, feed and fuel. TCP proteins are of particular interest for crop improvement programs due to their well-demonstrated roles in crop domestication and shaping plant architecture thereby, affecting agronomic traits. We identified 20 TCP genes from *Sorghum*. Except *SbTCP8*, all are either intronless or contain introns in the untranslated regions. Comparative phylogenetic analysis of *Arabidopsis*, rice, *Brachypodium* and *Sorghum* TCP proteins revealed two distinct classes categorized into ten sub-clades. Sub-clade F is dicot-specific, whereas A2, G1 and I1 groups only contained genes from grasses. Sub-clade B was missing in *Sorghum*, whereas group A1 was missing in rice indicating species-specific divergence of TCP proteins. TCP proteins of *Sorghum* are enriched in disorder promoting residues with class I containing higher percent disorder than class II proteins. Seven pairs of paralogous TCP genes were identified from *Sorghum*, five of which seem to predate Rice-*Sorghum* divergence. All of them have diverged in their expression. Based on the expression and orthology analysis, five *Sorghum* genes have been shortlisted for further investigation for their roles in regulating plant morphology. Whereas, three genes have been identified as candidates for engineering abiotic stress tolerance.

Manipulation of plant architecture has huge potential to improve the agronomic value of crop plants and has extensively been applied in domestication of grasses^1^. The molecular genetic characterization of the candidate genes, in the model systems rice and *Arabidopsis*, highlighted transcription factors as major players in shaping the plant morphology and architecture^2^,^3^.

With the discovery of the founding members, ***T**B1 (Teosinte Branched 1*) from maize, ***C**ycloidea* from snapdragon and ***P**CFs (Proliferation Cell Factors*) from rice, in the late 1990s, members of plant-specific TCP family of transcription factors, in particular, have been shown to play key roles in evolution of plant form and structure^4^. *TB1* controls vegetative axillary meristem outgrowth and played major role in domestication of modern maize from the ancestral teosinte^5^. *Cycloidea* of snapdragon regulates floral asymmetry, whereas, rice PCFs have been shown to promote cell proliferation and organ growth^6^,^7^.

Till date, more than 30 TCP genes have been characterized in plants, using reverse genetics approaches, to play key roles in regulating leaf size and curvature, lateral organ development, internode elongation, circadian rhythms, floral transition, floral symmetry, pollen development and senescence^8^,^9^,^10^,^11^,^12^. Recent findings in *Arabidopsis*, rice and tomato unleashed the role of TCP genes in regulating plant immunity and abiotic stress tolerance as well^13^,^14^. However, bulk of the characterization of TCP gene functions has been carried out in the model plant species *Arabidopsis*^11^. Apparently, with the huge diversity in plant morphology and forms in monocots and dicots, the complete picture of TCP gene function and regulation cannot be obtained based on the studies done in *Arabidopsis* alone. Therefore, identification and functional characterization of these genes in the plants of agronomic importance is required for harnessing their full potential in crop improvement programs.

*Sorghum* has recently emerged as an excellent model system for C4 grasses, especially bioenergy crops^15^. The multipurpose varieties of *Sorghum* have the potential to mitigate some of the challenges associated with food, feed, forage and fuel security. Because of wide range of genotypic and phenotypic diversity; identification and characterization of key genes regulating plant morphology and architecture in *Sorghum* is required to gain insights into the genetic basis of morphological agronomic traits.

In this study, we have leveraged publicly available genomic and transcriptomic resources to interpret TCP gene functions in *Sorghum*. Analysis of predicted gene structures, protein attributes and gene expression in developmental stages and abiotic stress-treated samples revealed targets for functional genomic studies. Furthermore, comparative phylogenomic analysis of TCP family transcription factors in *Arabidopsis* and three poaceae species, including rice, *Brachypodium* and *Sorghum*, provided insights into evolution and diversification of TCP gene functions. The shortlisted TCP genes, in this study, are promising targets for engineering morphological agronomic traits and stress tolerance in *Sorghum*.

## Results

### Identification, phylogeny and classification of TCP proteins in *Sorghum*

**Figure 1:**
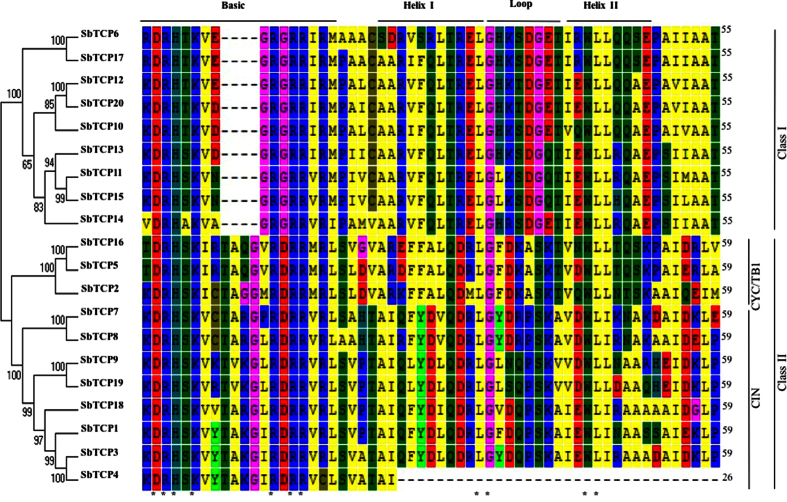
Multiple alignment of the TCP domains of *Sorghum* TCP proteins. The domain sequences were extracted and aligned using clustal X. The dendrogram on the right shows phylogenetic grouping of TCP proteins based on the complete protein sequences with bootstrap values given on each branch. The colors indicate amino acids of different biochemical properties as obtained through MEGA 6.0. The basic region, helix I and II, and loop regions are marked on the top. The classes, defined on the basis of phylogenetic tree are marked on the left. The conserved residues are marked by black asterisks at the base of alignment. The numbers on the right indicate the length of TCP domains in each protein.

Using HMM profile of the TCP proteins, we retrieved 20 TCP proteins from *Sorghum* proteome (https://phytozome.jgi.doe.gov/pz/portal.html). The detailed information about their Phytozome gene IDs, number of transcripts, length of proteins, and isoelectric point is provided in Table 1. All TCP proteins are characterized by a conserved non-canonical bHLH domain, known as TCP domain. The presence of domain was confirmed using InterProScan and protein sequences, corresponding to TCP domain, were extracted. Multiple alignment of the TCP domain sequences followed by phylogenetic analysis revealed two distinct classes of TCP proteins. Class I comprised of nine proteins, whereas, eleven proteins clubbed into class II. Class II was further subdivided into CYCLOIDEA/TB1 (CYC/TB1) clade comprising of three proteins and CINCINNATA (CIN) clade with eight proteins (Fig. 1).

Except for SbTCP4 that contained only partial TCP domain, basic, helix I, loop and helix II regions could be demarcated in all of the *Sorghum* TCP proteins. TCP domain in class I proteins possess a deletion of four amino acids in the basic region and therefore, basic region in class I proteins only comprises of 16 amino acids as opposed to 20 amino acids in class II proteins. Seven highly conserved residues including Asp (D), His (H), Lys (K) and four residues of Arg (R) characterize the basic region of all TCP proteins (Fig. 1). The abundance of Lys and Arg in basic region of TCP domain likely contributes to the nuclear localization of these proteins^4^. The helix I, loop and helix II regions comprised of 11, 8 and 9 amino acids, respectively. Two residues each in helix I (Ala and Leu) and helix II (Trp and Leu) were also highly conserved whereas, only one amino acid (Gly) was conserved in the loop region (Fig. 1).

### Genomic organization and duplication of *Sorghum* TCP proteins

**Figure 2:**
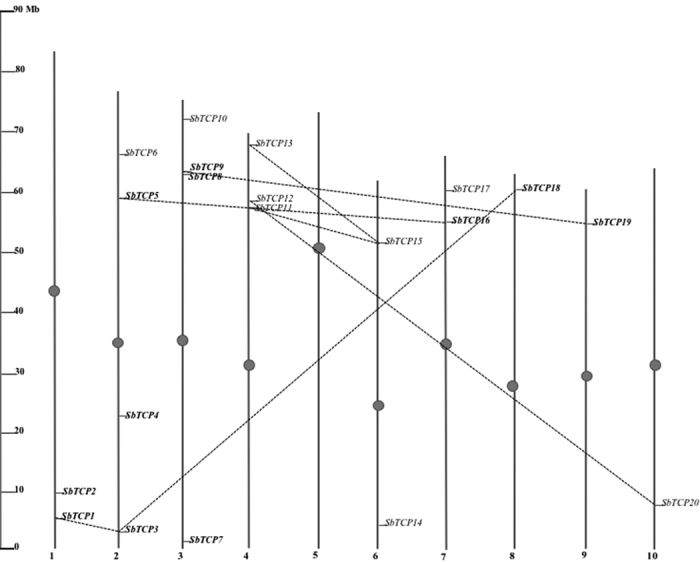
Chromosomal localization of duplicated TCP genes in *Sorghum*. TCP genes were mapped on the *Sorghum* chromosomes using genomic information available in Phytozome. The vertical bars represent the chromosomes with numbers at the base of each bar representing chromosome number. The ruler on the left indicates the length of the chromosomes in Mb. Class II genes are shown in bold. The duplicated genes pairs in the segmental duplicated blocks are connected by dotted lines.

The information about the genomic location of TCP genes was obtained from Phytozome and used to map TCP genes on the *Sorghum* chromosomes. TCP genes were spotted on all chromosomes of *Sorghum* except chromosome five. Chromosome 2 and 3 contained four TCP genes each, whereas, chromosome 4 contained three genes. Similarly, chromosomes 1, 6 and 7 contained two TCP genes each, whereas chromosomes 8, 9 and 10 had single TCP gene each (Fig. 2). All the TCP genes are well spaced on the *Sorghum* chromosomes with no tandem duplicates inferred. Based on their location on respective chromosomes, the genes were named as *SbTCP1*-*SbTCP20*.

To understand the pattern of expansion and diversification of TCP genes in *Sorghum*, duplicated genes were identified using plant duplication database. Seven pairs of possible paralogs of *TCP* genes were identified on the duplicated blocks of *Sorghum* genome. Among class I genes, duplicated genes could be marked on the duplicated segments of chromosomes 1 and 2 (*SbTCP1*/*SbTCP3*); chromosomes 2 and 8 (*SbTCP3*/*SbTCP18*); chromosomes 2 and 7 (*SbTCP5*/*SbTCP16*); and, chromosome 3 and 9 (*SbTCP9*/*SbTCP19*). Notably, *SbTCP3* is duplicated with both *SbTCP1* and *SbTCP18*. Among class II genes, duplicated genes were found on chromosomes 4 and 6 (*SbTCP13*/*SbTCP15* and *SbTCP11*/*SbTCP15*) and, chromosomes 4 and 10 (*SbTCP12*/*SbTCP20*). Again, *SbTCP15* is duplicated with *SbTCP11* as well as *SbTCP13* indicating common ancestry of these genes. The percentage similarity among duplicated genes ranged from 49.5 (*SbTCP1*/*SbTCP3*) to 70.2% (*SbTCP12*/*SbTCP20*) (Supplementary Table 1).

The number of synonymous (Ks) and non-synonymous (Ka) substitutions per site of the duplicated TCP genes in *Sorghum* were determined using tools available with DNA Sequence Polymorphism (DnaSP) software and Plant Genome Duplication Database (PGDD). The Ka/Ks values for all pairs of duplicated genes were <1 implying that duplicated gene pairs are under purifying selection. The Ks values were not used to further predict the time of duplication events of duplicated genes as all the TCP genes of *Sorghum* have an average GC content higher than 75% in the third codon position (Supplementary Table 2). The average GC content for *Sorghum* TCP genes in the third codon position is estimated to be 85.7%.

### Structural attributes of *Sorghum* TCP genes and proteins

**Figure 3:**
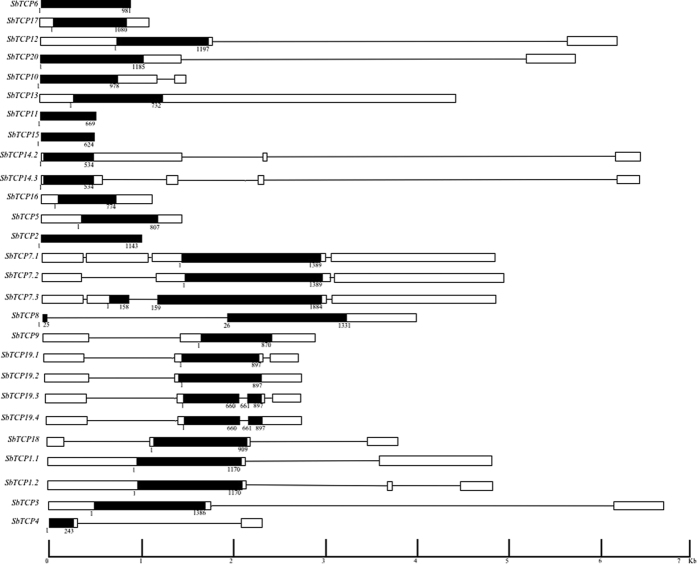
Structural organization of TCP genes in *Sorghum*. The black boxes represent coding region, whereas, white boxes represent 5′ and 3′ Untranslated regions (UTRs). The introns in coding region or UTRs are shown by black lines. The scale bar at the base represents the length of the genes in Kb. Numbers provided with the black boxes mark exon boundaries and represent the actual size of the coding region.

The length of the coding regions of TCP genes in *Sorghum* ranged from 243 (*SbTCP4*) to 1884 bp (*SbTCP8*) with an average length of 1071 bp. Analysis of intron-exon distribution revealed eight TCP genes of *Sorghum* as intronless, whereas eight contained one intron, three genes contained two introns and one gene (*SbTCP7*) contained three introns (Fig. 3). In class II, all three genes belonging to CYC/TB1 clade were intronless, whereas those comprising CIN clade had at least one intron. Length of the introns in these genes ranged from 101 to 4341 bp. Out of 12 genes containing intron(s), 11 had introns in the untranslated regions (UTRs). Among these, one gene had intron in the 5′UTR and seven in the 3′UTR, whereas three genes had introns in both 5′ and 3′ UTRs (Fig. 3). Only one gene, *SbTCP8* possessed intron in the coding sequence.

Four TCP genes of *Sorghum*, one from class I (*SbTCP14*) and three from class II (*SbTCP1, SbTCP7* and *SbTCP19*) were predicted to encode more than one transcript (gene models; Table 1) due to alternative splicing. However, except for one of the gene models of *SbTCP7*, all other gene models for the four genes encode for the same protein indicating that introns in the untranslated regions of these genes led to splicing variants (Fig. 3).

The length of the proteins ranged from 80 (*SbTCP4*) to 627 (*SbTCP7*) amino acids with an average of 325 amino acids. Earlier study in *Arabidopsis* reported that amino acid composition of TCP family proteins is typical of intrinsically disordered proteins with both class I and II proteins enriched in disorder promoting residues viz., Gln, Asn, Pro and Ser^16^. Analysis of TCP proteins of *Sorghum* with PONDR VL-XT predictor predicted degree of disorder ranging from 44.3 to 91.25% in these proteins (Table 1). The number of disordered regions in *Sorghum* proteins varied from 1 to 16. All the class I proteins showed high percent disorder (≥60%) compared to class II proteins where only four proteins had disorder percentage higher than 60%.

### Predicted subcellular localization

Subcellular localization of TCP proteins was predicted using six different tools and an overall prediction was made by compiling the high confidence predictions from these tools (Table 1 and Supplementary Table 3). Ten of the *Sorghum* TCP proteins were predicted to localize in the nucleus. Two proteins, SbTCP4 and 14 were predicted to localize in the chloroplasts, whereas SbTCP10 was predicted to localize in the cytoplasm. The localization of SbTCP15 was predicted in both nucleus and chloroplast, suggesting that it may be required for coordinated expression in both nuclear and chloroplast compartments.

### Expression analysis of *Sorghum* TCP genes

**Figure 4:**
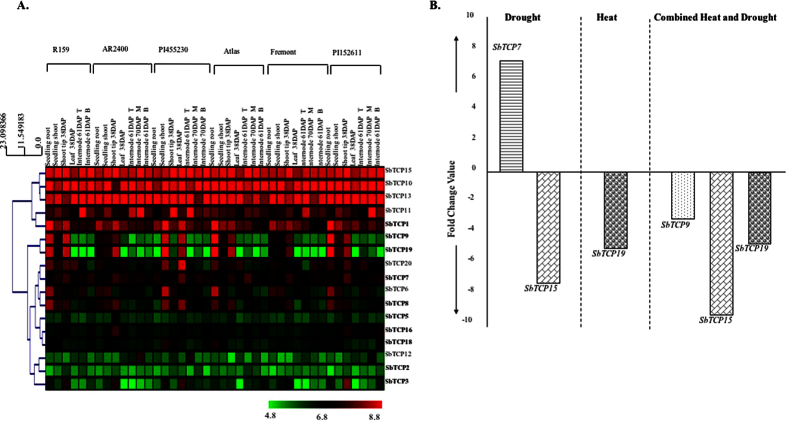
Expression analysis of TCP genes in vegetative tissues and in response to abiotic stress treatments. (**A**) Expression analysis in vegetative tissues from diverse genotypes of *Sorghum*. Expression profile in six different genotypes of *Sorghum* in seedling root, seedling shoot, shoot tip, leaf and, stem internodes. The dendrogram on the left represents hierarchical clustering based on expression values. (**B**) Differential accumulation of TCP genes in response to heat, drought and combined stresses. The Y-axis represents the average fold changes between three biological replicates. Only the genes exhibiting more than two-fold change in all three replicates are shown.

We used publicly available microarray-based (Affymetrix platform) expression data from six genotypes in five vegetative tissues including seedling root, seedling shoot, shoot tip, leaf and stem internodes for analyzing TCP gene expression. Out of 20 TCP genes, 17 are represented on the Affymetrix arrays (Fig. 4A). The expression data clearly revealed high-level ubiquitous expression of four class I genes, namely *SbTCP5, 10, 11* and *13*, in all the stages and genotypes. Whereas, *SbTCP6* and *20* of class I exhibited dominant expression in seedling roots, shoots and shoot tips. Transcripts of *SbTCP12* were specifically detected in limited portions of internodes of three bioenergy/high biomass lines, PI455230, PI152611 and AR2400. Genotype-specific variation in the expression pattern of TCP genes indicates differential activity of TCP genes in genotypes exhibiting different morphological features.

Among class II genes, we noticed predominant expression of three genes including *SbTCP8, 9* and *19* in tissues undergoing active cell division and elongation such as seedling shoots, roots and shoot tips. Whereas high expression of *SbTCP1* and *7* was detected in most of the tissues analyzed. *SbTCP5* expression was only detected in shoot tips, whereas *SbTCP3* seemed to exhibit variable expression pattern in different genotypes mostly in shoot tip, seedling shoots and leaf tissue. We could not detect significant expression of *SbTCP2* in any of the analyzed stages.

Further, the expression of all TCP genes was examined in microarray-based (Agilent platform) expression data in response to heat, drought and, combined heat and drought stress in *Sorghum*. Expression of only four TCP genes, *SbTCP7, 9, 15* and *19*, was affected by at least one of these stress treatments (Fig. 4B). *SbTCP7* was the only gene upregulated in response to drought stress. *SbTCP15* was downregulated in response to drought and combined stresses but remained unaffected by heat stress alone. Whereas *SbTCP19* was downregulated by heat and combined stress but remained unaffected by drought stress; *SbTCP9* was downregulated by combined stress but remained unaffected in response to individual stresses.

**Figure 5:**
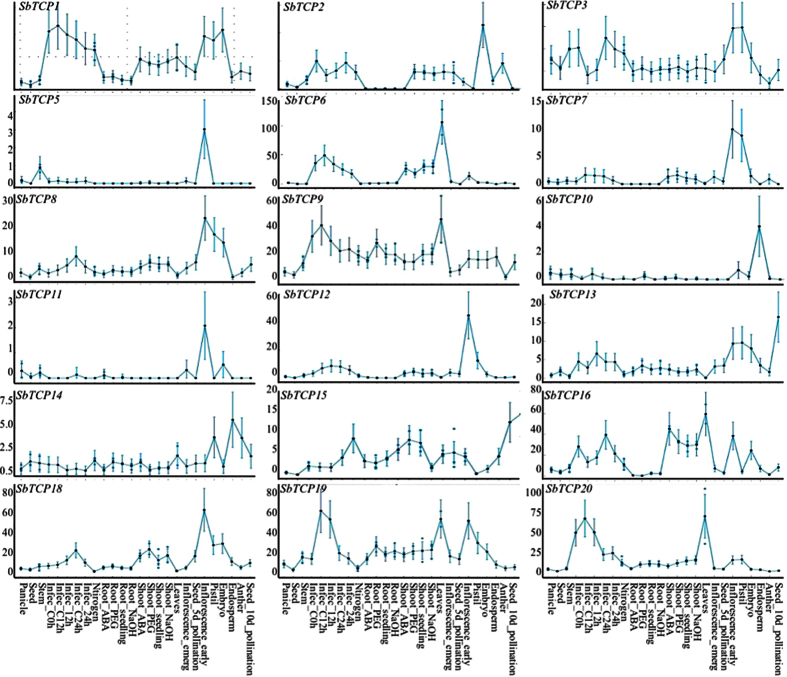
RNAseq based expression patterns of *Sorghum* TCP genes. Expression profiles for all the *Sorghum* TCP genes were downloaded from Morokoshi database. The X-axis represents the different stages of development or stress conditions and the Y-axis represents FPKM (fragments per Kb per million) values for TCP transcripts.

Furthermore, to analyze the expression of TCP genes in reproductive tissues and in response to biotic stress treatments, we queried RNA Sequencing (RNAseq) based expression data available in MOROKOSHI *Sorghum* Transcriptome database (http://sorghum.riken.jp/morokoshi/Home.html). Out of 20 TCP genes, 18 were present in MOROKOSHI database. Except for two class I genes, *SbTCP10* and *20*, which exhibited high accumulation in vegetative tissues, high-level expression of the rest 16 genes was detected in reproductive tissues. Overall, class I genes mostly exhibited high expression in vegetative tissues and early floral developmental stages, whereas class II genes were mainly detected in floral organs and seed tissues. In agreement with the previous observation, expression of *SbTCP7* and *19* was affected by abiotic stress treatments. However, no obvious induction in response to infection with *Bipolaris sorghicola* was detected for any of the TCP genes (Fig. 5). Taken together, all of the *Sorghum* TCP genes exhibit high level expression in at least one of the analyzed stages except *SbTCP4, 14* and *17* which were neither represented on chip nor detected in the RNAseq data.

A comparison of expression patterns of duplicated genes *SbTCP8* and *19* showed a highly similar expression pattern in vegetative tissues with correlation coefficient >0.9. However, both the paralogs exhibited different expression profiles in inflorescence stages indicating functional divergence in reproductive tissues between them (Fig. 5.). The correlation coefficient in expression patterns of the rest of the duplicated gene pairs range from −0.10 for *SbTCP13*/*SbTCP15* to 0.72 for *SbTCP5*/*SbTCP16* in vegetative tissues suggesting subfunctionalization or neofunctionalization as the most common fate of duplicated TCP genes in *Sorghum*. We blasted *Sorghum* orthologs in rice followed by reverse blast analysis to check if duplicated gene pairs predicted in *Sorghum* have arisen before Rice-*Sorghum* divergence. Five of the TCP gene duplications seem to predate Rice-*Sorghum* divergence with *Sorghum* paralogs *SbTCP9*/*19* orthologous to rice paralogous genes *OsTCP5*/*18; SbTCP5*/*16* paralogs of *Sorghum* orthologous to *OsTCP22*/*24* of rice; *SbTCP1*/*3* paralogs orthologous to rice *PCF6*/*OsTCP21; SbTCP11*/*15* paralogs orthologous to rice *OsTCP7*/*17* and *SbTCP12*/*20* paralogs orthologous to rice *OsTCP9*/*19* gene pairs (Supplementary Table 4).

### Comparative phylogenetic and structural analysis of TCP proteins in *Arabidopsis* and grasses

**Figure 6:**
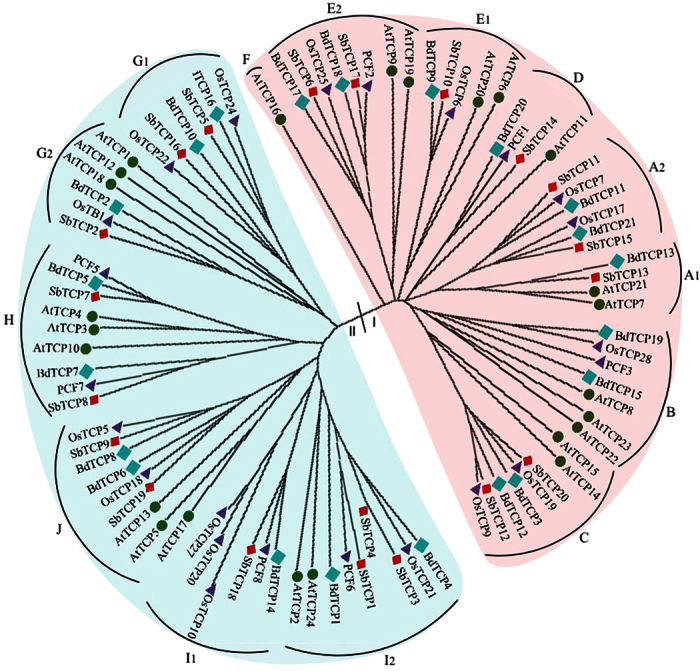
Phylogenetic relationship among TCP proteins in *Arabidopsis*, rice, *Brachypodium* and *Sorghum*. Full length amino acid sequences of TCP proteins of *Arabidopsis*, rice, *Brachypodium* and *Sorghum* were used to generate an unrooted Neighbor-joining radial tree constructed with 1000 bootstrap iterations. Different colors have been used to highlight class I and II proteins. Sub-clades have been marked in each class. Different symbols have been used to differentiate TCP prteins from different species.

In order to understand the evolutionary relationships among TCP proteins of *Arabidopsis* and grasses, we constructed an un-rooted neighbor joining phylogenetic trees using 88 complete protein sequences of TCP genes from *Arabidopsis* and three poaceae species (Fig. 6). These included 23 rice, 24 *Arabidopsis*, 21 *Brachypodium* and 20 *Sorghum* TCP proteins. The TCP proteins from these species could be categorized into two distinct classes, class I and class II. Class I comprised of 43 proteins including 10 rice, 9 *Sorghum*, 13 *Arabidopsis* and 11 *Brachypodium* proteins. Class I further could be subdivided into six sub-clades, named A, B, C, D, E and F. Sbu-clades A, B, D and E comprised of members from all four species. Sub-clade C however, was specific to monocots with no protein from *Arabidopsis*, whereas sub-clade F contained a single TCP protein AtTCP16 of *Arabidopsis* with no protein from grass genomes.

Class II comprised of two major clades including CYC/TB1 and CIN clade. CYC/TB1 clade, containing single sub-clade G comprised of twelve genes with three genes each from all four species. Two distinct groups, named G1 and G2, were apparent in sub-clade G. The group G1 only comprised of monocot proteins including rice OsTCP22 and 24 (Retarded Palea 1); *Brachypodium* BdTCP10 and 16; and *Sorghum* SbTCP5 and 16. The Retarded Palea 1 (REP1) protein of rice in monocot-specific group G1 regulates palea development and floral zygomorphy^17^, characteristic to monocot species, indicating divergence of G1 group to regulate monocot-specific morphological features. Whereas, G2 comprised of three proteins of *Arabidopsis* (AtTCP1, 12 and 18) and one protein each from rice (OsTB1), *Brachypodium* (BdTCP2) and *Sorghum* (SbTCP2).

CIN clade of class II proteins comprised of 33 proteins with seven *Brachypodium*, ten rice, eight *Sorghum* and eight *Arabidopsis* proteins. These were organized into three distinct sub-clades named H, I and J, with each of them containing members from all four species. However, as expected, the TCP proteins from monocot species were more closely related to each other compared to the *Arabidopsis* orthologs.

Further, we used MEME motif search tool to identify the conserved motifs besides TCP domain in TCP proteins from all four species (Supplementary Fig. 1). A total of twenty motifs of lengths ranging from 6 to 57 amino acids were identified and their distribution in all TCP proteins was compared. The assignment of motifs reiterated the phylogenetic analysis and enabled a clear classification of class I and class II TCPs based on the motifs present in the two classes, indicating that similar motifs render similar functionalities to each class. Motifs 1, 2 and 3 represent TCP domains. All the class I proteins, except BdTCP13, possessed motif 1, whereas TCP domain in class II proteins, except SbTCP4, was represented by the motif 2. Motif 3 was present in all class I proteins and one class II protein namely, SbTCP4. Motif 9, representing arginine rich R domain, was specific to class II proteins and detected in thirteen proteins (BdTCP4, OsTCP21, SbTCP3, AtTCP24, AtTCP2, OsTCP10, OsTCP20, SbTCP2, OsTB1, BdTCP2, AtTCP18, AtTCP12 and AtTCP1). Whereas, motif 12, representing the target region of miR319, is specific to class II proteins and was detected in 15 TCP proteins (BdTCP4, OsTCP21, SbTCP3, SbTCP1, PCF6, BdTCP1, AtTCP24, AtTCP2, BdTCP14, PCF8, SbTCP18, AtTCP3, AtTCP4, BdTCP5 and PCF5) indicating the possibility of miRNA-mediated regulation of class II proteins. In addition to these motifs, we detected SP domain (rich in serine and proline residues), (PSVKHMFPFCDSSSPMDLPLYQQLQLSPPSPKPD), characteristic of TB1 like proteins in SbTCP2 and OsTB1^18^ as well as coiled coil (CC) domain (AEPSIIAATGTGVTP) in eight class I (PCF1, AtTCP20, AtTCP11, AtTCP21, AtTCP7, BdTCP20, SbTCP13, SbTCP14) and six class II (PCF2, OsTCP28, AtTCP23, AtTCP9, SbTCP6, SbTCP17) proteins. The functional relevance of these domains is yet to be determined.

### Comparative expression profiling of TCP genes in *Sorghum*, rice and *Brachypodium*

**Figure 7:**
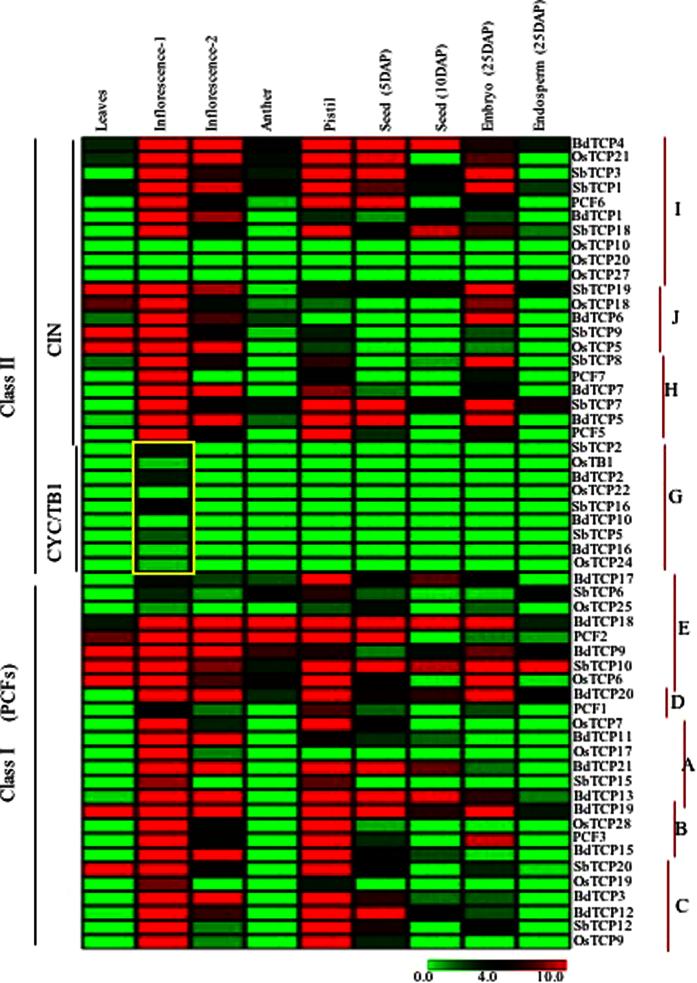
Comparative expression analysis of TCP genes in rice, *Brachypodium* and *Sorghum*. Heat map was generated based on FPKM values obtained from the previously published transcriptomic data^20^. The scale bar at the base represents relative expression values.

Earlier, we had compared the expression of TCP genes in rice and *Arabidopsis* by analyzing their expression patterns in similar stages of development^19^. To further investigate the conservation and/or diversification in the expression patterns of TCP genes in grasses, we leveraged the RNA sequencing based expression data available from physiologically similar tissues of three grass species representing three different lineages including the Pooideae (*Brachypodium*), Panicoideae (*Sorghum*) and Ehrhartoideae (rice) of family poaceae^20^. The comparative expression profiles of all the genes are illustrated in the order of their phylogenetic placement in the comparative phylogenetic tree (Fig. 7).

In sub-clades A-E, we noticed that rice transcripts were mostly expressed in inflorescence and pistil tissues, whereas orthologous genes from *Brachypodium* and *Sorghum* were more broadly expressed with many of them expressing in seed tissues as well.

Sub-clade G includes CYC/TB1-like genes from all three species. Genes from all three species in this sub-clade exhibit low level but highly specific expression restricted to primordial inflorescence. Sub-clade H genes from all three species expressed in inflorescence, pistils and embryos. Transcripts of *BdTCP4, OsTCP21, SbTCP1* and *SbTCP3* of sub-clade I were detected in anthers, inflorescence, pistils and seed tissues. However, no expression was detected from *OsTCP10, 20* and *27* genes of rice in any of the analyzed stages.

Sub-clade J genes were expressed in both leaves and reproductive tissues including inflorescence and embryos, whereas expression of sub-clade H genes was restricted to reproductive tissues. Overall, sub-clade G genes seemed to have highly conserved role in the primorida inflorescence in all three species, whereas, expression patterns of the genes in rest of the sub-clades seem to have diversified in all three species.

## Discussion

*Sorghum* is a photosynthetically efficient C4 crop cultivated in wide range of agro-ecological conditions across 98 countries of the world^21^. It not only feeds over 750 million people in semi-arid tropical regions of Africa, Asia and Latin America^22^ but is also used as forage for dairy cattle and, has recently emerged as a promising feedstock for biofuels^15^. However, *Sorghum* varieties exhibit great deal of genotypic and phenotypic diversity in the field conditions. Based on the morphological and agronomic traits, *Sorghum* cultivars have been categorized into four ideotypes namely, grain, forage, energy and sweet *sorghum*. Interestingly, some of their morphological traits are directly associated with one or more agronomically important traits. For example, brown midrib phenotype is associated with the fodder quality in forage *sorghum*^23^, whereas green color of midrib and length of internodes are correlated with the sugar yield, juice volume and moisture in sweet *sorghum*^24^,^25^. Similarly, grain yield in grain *Sorghum* is affected by inflorescence exertion, flowering time, shape and size of the panicle^15^. Moreover, resistance to biotic and abiotic stresses may also be associated with plant morphology in *Sorghum*. For example, the genotypes with closed glumes have been shown to exhibit enhanced resistance to grain mold^26^. Therefore, directed modification of plant morphology in *Sorghum* can have profound effect on yield and varietal improvement.

Members of TCP gene family have been recruited to generate novel morphological traits during plant evolution and therefore, provide an important resource to generate desired phenotypes. *TB1* of maize, a TCP family transcription factor, is a classic example demonstrating role of a single gene in crop domestication^5^. *Sorghum* ortholog of maize *TB1, SbTB1* has also been implicated in regulating axillary bud outgrowth in response to light signals^27^. However, to realize the full potential of TCP genes in engineering *Sorghum* varieties and shortlist targets for engineering, a genome-wide analysis of TCP family in *Sorghum* was needed. Here, in this study, we provide a comprehensive overview of TCP gene family in *Sorghum* and based on the comparative phylogenomic analysis in *Arabidopsis* and three poaceae species short list candidates for experimental verification.

### *Arabidopsis* and grass-diverged aspects of TCP family evolution

The number of TCP genes identified in *Sorghum* (20) is similar to that of *Arabidopsis*^10^ (24), rice^19^ (23) and *Brachypodium* (21). This result is consistent with the earlier observations that these species possess a similar number and sizes of gene families^28^,^29^. Domain alignment and phylogenetic analysis revealed similar organization with two distinct classes I and II of TCP proteins in *Sorghum* like that of rice and *Arabidopsis*. Among class II proteins, two distinctive clades, earlier named as CYC/TB1 and CIN clades in rice and *Arabidopsis*^10^, were identified. To infer the functions of the genes in these clades, we performed a comparative phylogenetic analysis of TCP genes in *Arabidopsis* and three poaceae species and employed classification scheme proposed earlier in cotton^30^ to classify them into sub-clades A to J. Members of all four species were identified from all the sub-clades except sub-clade F which seems to be specific to dicots and sub-clade B that seems to be missing in *Sorghum*.

On the other hand, in A1 group of sub-clade A, no rice protein was found. This group contained two *Arabidopsis* proteins (AtTCP7 and 21), one *Sorghum* protein (SbTCP13) and one *Brachypodium* protein (BdTCP13). AtTCP21, also known as CCA1 Hiking Expedition 1 (CHE1), is an important circadian regulator in *Arabidopsis*^31^. Orthology analysis using reverse blast analysis indicate that *SbTCP13* and *BdTCP13* in this group are orthologous to *Arabidopsis* AtTCP7, whereas CHE1 orthologs are missing in these species.

Conversely, three groups including group A2 of sub-clade A, group G1 of sub-clade G and I1 of sub-clade I only contain genes from grasses with no *Arabidopsis* ortholog. These genes seem to have arisen to cater monocot-specific traits. For example, *Retarded Palea 1 (REP1*) gene of rice controls palea development and floral zygomorphy in rice^17^. Floral zygomorphy is characteristic to all three poaceae species including rice, *Sorghum* and *Brachypodium*, whereas *Arabidopsis* has radially symmetrical flowers. Consistent with this observation, group G1 containing REP1 had no ortholog in *Arabidopsis* suggesting that this group has diverged to regulate floral asymmetry in grasses. Further characterization of monocot-diverged genes in these groups may provide candidates for improving agronomic potential of grasses.

Genome duplication seems to have played major role in the expansion of TCP family in higher plants. Seven pairs of paralogous genes were identified from *Sorghum* and an equal number of TCP gene pairs have been earlier reported from duplicated segments of the rice genome^19^. Most of the duplicated gene pairs detected in *Sorghum* seem to predate *Rice*-*Sorghum* divergence. However, these seem to have diverged in their structure and expression patterns indicating neo/subfunctionalization as major fate of duplicated genes in TCP family of *Sorghum*. Further, less than 1 Ka/Ks ratios between paralogous genes indicate purifying/stabilizing selection on the retained duplicates and their importance for the fitness of the plant.

### Structure and expression of TCP genes indicate an inherent property as important transcriptional regulators during cell division

Structural analysis revealed presence of introns mainly in the UTR regions of TCP genes^19^ and a conserved motif representing target of miRNA-mediated posttranscriptional regulation^11^, indicating enhanced robustness to transcriptional or missplicing-related errors in these genes^32^. Moreover, all *Sorghum* TCP genes, except SbTCP7.3 and SbTCP8, lack introns in the coding regions which is generally associated with rapid regulation during cell division and cell differentiation^33^,^34^.

Furthermore, similar to reports in *Arabidopsis*, TCP proteins of *Sorghum* are also rich in disorder promoting residues. C-terminal intrinsically disordered region in *Arabidopsis* TCP8 has been shown to act as transactivation domain^16^. Presence of disordered regions and transactivation domains in TCP proteins indicate their activity as autonomous factors for activating transcriptional machinery and fine regulation during adaptive responses where gene expression would depend on tissue/stimulus-specific interactions.

The expression patterns of TCP genes also correlate well with their roles in regulating cell proliferation in meristematic tissues^7^. Expression analysis revealed high expression of *Sorghum* TCP genes in tissues undergoing active cell division such as seedlings shoots, roots, shoot tips and internodes, which is consistent to the reports in rice and *Arabidopsis*^19^. During reproductive development also, TCP genes usually express at high levels in early stages of inflorescence development.

### *Sorghum* TCP candidates for engineering plant morphology and abiotic stress tolerance

TCP genes in model systems, rice and *Arabidopsis* have been shown to play key roles in integrating hormonal, environmental and developmental signals to regulate plant development and morphology^11^. Characterization of target genes in *Sorghum* will not only assist in modeling *Sorghum* varieties for desirable attributes but will also add another missing piece to the molecular genetic jigsaw puzzle of gene-environment interactions affecting morphological traits in crop plants.

*SbTCP7* of *Sorghum* is orthologous to rice *PCF5* and is the only gene upregulated in response to drought stress in our study. Negative regulation of *PCF5* through miR319 confers increased tolerance against drought and salinity stress in rice^35^,^36^. MEME motif analysis revealed that *SbTCP7* contained the motif 12 that corresponds to miR319 binding site, suggesting that *SbTCP7* might also be involved in regulation of abiotic stresses in *Sorghum* through similar mechanism as in rice.

Another gene pair *SbTCP9* and *19* of sub-clade J is orthologous to *OsTCP5* and *OsTCP18* of rice and were predominantly detected in meristematic tissues such as seedlings shoots, roots, shoot tips and leaves. Although nothing is known about *OsTCP18* of rice, *OsTCP5* has been shown to be regulated by strigalactones and cytokinins and is implicated in regulating mesocotyl length^37^. *Arabidopsis* orthologs (*AtTCP5, 13* and *17*) of these genes regulate cell proliferation and differentiation during leaf development^38^. When overexpressed, these conferred enhanced drought and salinity tolerance in *Arabidopsis*^39^. Both the *Sorghum* genes *SbTCP9* and SbTCP19 were also downregulated by abiotic stress treatments indicating a possible involvement in abiotic stress response.

Furthermore, preferential location of introns in 5′ and 3′ UTR regions in all three genes, *SbTCP7, 9* and *19*, might not be circumstantial. Presence of introns in UTRs is a classic feature of substrates for non-sense mediated decay and posttranscriptional regulation^40^. *SbTCP7* and *19* also exhibit alternative splicing which is an important component of stress response by regulating choice of splice sites^41^.

Based on the expression and orthology analysis, *Sorghum* genes of CYC/TB1 clade (*SbTCP2, 5* and *16*) in particular are good candidates for engineering shoot branching and panicle morphology. *SbTCP2* is orthologous to rice *TB1* that regulates tillering in rice^18^,^42^ and *SbTCP5* is ortholog of rice *REP1* involved in regulating palea development and floral zygomorphy^17^. Duplicated genes, *SbTCP1* and *3* are orthologous to paralogous genes *AtTCP2* and *24* of *Arabidopsis* and, *PCF6* and *OsTCP21* of rice. Both the genes in *Arabidopsis* and rice are regulated by miR319 and implicated in organ development and cold stress response, respectively. Both *SbTCP1* and *3* contain target region of miR319 (motif 12) indicating conserved miRNA-mediated regulation of the orthologous genes in *Sorghum* as well.

## Methods

### Identification, chromosomal localization and duplication analysis

The Hidden Markov Model (HMM) profile for the TCP domain was generated from 1704 TCP proteins downloaded from the Plant Transcription Factor Database (http://planttfdb.cbi.pku.edu.cn/)^43^ using HMMER V3.1b1 (http://hmmer.org/)^44^. Complete proteome of *Sorghum* was downloaded from Phytozome (http://www.phytozome.net/)^45^. Both in-house generated HMM profile and one (PF03634) downloaded from Pfam Database (http://pfam.xfam.org/)^46^ were independently used as a query to perform HMMER search against *Sorghum* proteome using default parameters with e-value cut off set to 0.01. The list of TCP proteins of rice and *Arabidopsis* was obtained from the previous studies^19^ and protein sequences were downloaded from rice genome annotation project database (http://rice.plantbiology.msu.edu/)^47^ and The *Arabidopsis* Information Resource (https://www.arabidopsis.org/)^48^ database, respectively. The TCP sequences for *Brachypodium* were downloaded from Grassius database (http://grassius.org/grasstfdb.html)^49^. To verify the presence of TCP domain, we scanned all the protein sequences using InterProScan (https://www.ebi.ac.uk/interpro/search/sequence-search). The physical location of the *Sorghum* TCP genes was obtained from Phytozome (http://www.phytozome.net/) and used to localize TCP genes on respective chromosomes. Following the convention used in other species, the genes were named as *SbTCP1* to *SbTCP20*, based on their location on the respective chromosomes. The information about duplicated genes was obtained from Plant Genome Duplication database (PGDD, http://chibba.agtec.uga.edu/duplication/). Both PGDD and DnaSp software were used to calculate rate of non-synonymous substitutions (Ka) and synonymous substitutions (Ks).

### Phylogenetic analysis and identification of orthologs

Multiple sequence alignment of TCP proteins of *Sorghum*, rice, *Arabidopsis* and *Brachypodium* was performed using online Fast Fourier Transform tool (MAFFT) http://www.ebi.ac.uk/Tools/msa/mafft/)^50^ with local pair fast Fourier transformation and default parameters. We used MEGA 6.06 software (http://www.megasoftware.net/)^51^ to build the neighbor-joining phylogenetic tree from the sequence alignment using following parameters: p-distance model, pairwise gap deletion and 1000 bootstraps. A separate tree was constructed with only TCP proteins of *Sorghum* using same parameters. Further, to determine the homologous relationships among characterized TCP proteins of rice and *Arabidopsis* with that of *Sorghum* TCP proteins, we blasted *Sorghum* TCP proteins against rice and *Arabidopsis* proteomes downloaded from RGAP and TAIR databases, respectively. The first five hits with percent similarity higher than 30%, E-value 1e-5 and score cut off of 100 were reverse blasted against *Sorghum* proteome. If the same *Sorghum* protein that was queried in rice and *Arabidopsis* was obtained among the first three hits in *Sorghum* proteome, the genes were predicted to have orthologous relationship.

### Gene structure analysis and identification of conserved motifs

The information about the gene length and distribution of exons, and introns in TCP genes was obtained from Phytozome. The Intron/Exon organization for TCP genes was determined by aligning the cDNA sequences to their corresponding genomic DNA sequences and using the result as the input for graphical display at the Gene Structure Display Server v2 (http://gsds.cbi.pku.edu.cn/). The intron/exon organization in UTR regions was checked manually.

Further, to identify the conserved motifs in TCP proteins, the online Multiple Expectation-Maximization for Motif Elicitation (MEME) (http://meme-suite.org/tools/meme)^52^ program was used using following parameters: length of motif minimum 6 and maximum 100. And maximum number of motifs set to 20.

### Subcellular localization and disorder predictions

To predict the subcellular localization of TCP proteins, we used six independent prediction databases including Plant mPLoc (http://www.csbio.sjtu.edu.cn/bioinf/plant-multi/)^53^, WoLFPSORT (http://www.genscript.com/wolf-psort.html)^54^, DISTILL (http://distill.ucd.ie/distill/)^55^, MultiLoc2 (http://abi.inf.uni-tuebingen.de/Services/MultiLoc2), SubLoc v1.0 (http://www.bioinfo.tsinghua.edu.cn/SubLoc/)^56^ and CELLO v2.5 (http://cello.life.nctu.edu.tw/)^57^. Except for Plant-mPloc, all other prediction programs provide confidence scores. The cutoff used to select high confidence predictions from WoLFPSORT, MultiLoc2, SubLoc v1.0 and CELLO v 2.5 were confidence score >12, confidence score >0.7, accuracy >95% and reliability score >4.0, respectively. For DISTILL predictions also, only high confidence results were considered. The final prediction was made after compiling only the high confidence predictions from these databases.

The disordered regions in TCP proteins were predicted using VLXT method in online Predictor of Natural Disordered Regions (PONDR^®^, http://www.pondr.com/). VLXT integrates all three feedforward neural networks including VL1 predictor, N-terminus predictor (XN) and the C-terminus predictor (XC) trained using disordered regions of varying length.

### Expression analysis

For expression analysis of TCP genes, we used publicly available microarray and RNA Seq-based expression data for *Sorghum*. For expression analysis during vegetative stages of development, we used Affymetrix data submitted under accession number GSE49879 at NCBI-GEO^58^. Similarly, we used data generated in response to individual and combined heat and drought stresses using Agilent 28 K array^59^ submitted under accession number GSE48205 for expression analysis of TCP genes in response to abiotic stresses. We also downloaded expression profiles of all *Sorghum* TCP genes from MOROKOSHI database (http://sorghum.riken.jp/morokoshi/Home.html)^60^. For comparative transcriptomic analysis of TCP family genes in rice, *Brachypodium* and *Sorghum*, we used the already available RNAseq data generated in physiologically similar tissues of these species^20^.

## Additional Information

**How to cite this article**: Francis, A. *et al*. Comparative phylogenomic analysis provides insights into TCP gene functions in *Sorghum. Sci. Rep.* **6**, 38488; doi: 10.1038/srep38488 (2016).

**Publisher's note:** Springer Nature remains neutral with regard to jurisdictional claims in published maps and institutional affiliations.

## Supplementary Material

Supplementary Dataset 1

## Figures and Tables

**Table 1:** TCP genes in *Sorghum* and their key features.

S. No.	Accession IDs	Gene Name	Type	No. of Transcripts	Protein Length (aa)	Isoelectric Point (pI)	Predicted Subcellular Localization	Number of Predicted Disordered Regions	Percent Disordered Region
1	Sobic.001G066100	*SbTCP1*	Class II	2	389	9.12	Nuclear	8	46.27
					389	9.12			
2	Sobic.001G121600	*SbTCP2*	Class II	1	380	8.5	Nuclear	5	70.79
3	Sobic.002G035500	*SbTCP3*	Class II	1	461	9.45	Nuclear	10	49.67
4	Sobic.002G141450	*SbTCP4*	Class II	1	80	10.03	Chloroplast	1	91.25
5	Sobic.002G198400	*SbTCP5*	Class II	1	268	5.82	Nuclear	5	65.67
6	Sobic.002G268600	*SbTCP6*	Class I	1	326	5.17	—	3	86.81
7	Sobic.003G018700	*SbTCP7*	Class II	3	462	6.46	Nuclear	13	48.48
					462	6.46			
					627	9.13		16	48.96
8	Sobic.003G299700	*SbTCP8*	Class II	1	451	6.62	Nuclear	10	52.33
9	Sobic.003G305000	*SbTCP9*	Class II	1	289	6.81	—	6	45.33
10	Sobic.003G408400	*SbTCP10*	Class I	1	325	6.34	Cytoplasmic	6	64
11	Sobic.004G225400	*SbTCP11*	Class I	1	222	9.9	—	4	59.46
12	Sobic.004G237300	*SbTCP12*	Class I	1	398	9.42	Nuclear	7	69.1
13	Sobic.004G354700	*SbTCP13*	Class I	1	243	10.09	—	5	62.14
14	Sobic.006G025000	*SbTCP14*	Class I	3	177	8.11	Chloroplast	3	87.01
					177	8.11			
15	Sobic.006G154000	*SbTCP15*	Class I	1	207	9.77	Nuclear/Chloroplast	4	72.95
16	Sobic.007G135700	*SbTCP16*	Class II	1	257	6.23	Nuclear	6	66.15
17	Sobic.007G182101	*SbTCP17*	Class I	1	359	5.71	Nuclear	5	74.93
18	Sobic.008G172200	*SbTCP18*	Class II	1	302	6.59	—	7	50.66
19	Sobic.009G195000	*SbTCP19*	Class II	4	298	7.94	—	8	44.3
					298	7.94			
					268	7.9			
					268	7.9			
20	Sobic.010G092100	*SbTCP20*	Class I	1	394	8.91	Nuclear	8	61.93
