# Corrigendum: Comparative phylogenomic analysis provides insights into TCP gene functions in *Sorghum*

**DOI:** 10.1038/srep45801

**Published:** 2017-04-06

**Authors:** Aleena Francis, Namrata Dhaka, Mohit Bakshi, Ki-Hong Jung, Manoj K. Sharma, Rita Sharma

Scientific Reports 6: Article number: srep3848810.1038/srep38488; published online: 11 05 2016; updated: 04 06 2017

The Acknowledgments section of this Article is incomplete, where:

“R.S. and M.K.S. acknowledge the Ramalingaswami fellowship and grant from the Department of Biotechnology, Government of India”.

should read:

“R.S. and M.K.S. acknowledge the Ramalingaswami fellowship and grant from the Department of Biotechnology, Government of India. Authors also acknowledge the financial support through PURSE grant from Department of Science & Technology, Government of India”.

