# Supplementary material for: Comparative phylogenomic analysis provides insights into TCP gene functions in Sorghum
Source: Sci Rep. 2016 Dec 5;6:38488. doi: 10.1038/srep38488 (PMC5137041; doi:10.1038/srep38488)
Supplement: Supplementary Dataset 1 [file srep38488-s1.pdf]

# Supplementary Data

**Title: Comparative phylogenomic analysis provides insights into TCP gene functions in *Sorghum***

Aleena Francis[1], Namrata Dhaka[1], Mohit Bakshi[1], Ki-Hong Jung[2], Manoj K. Sharma[3] and Rita Sharma[1,*]

**Supplementary Table 1.** The percentage similarity among duplicated TCP proteins of *Sorghum.*

| S. No. | Protein 1 | Protein 2 | Percent Identity |
|---|---|---|---|
| 1 | SbTCP1 | SbTCP3 | 49.51 |
| 2 | SbTCP3 | SbTCP18 | 58.65 |
| 3 | SbTCP5 | SbTCP16 | 65.22 |
| 4 | SbTCP9 | SbTCP19 | 51.81 |
| 5 | SbTCP11 | SbTCP15 | 66.47 |
| 6 | SbTCP12 | SbTCP20 | 70.22 |
| 7 | SbTCP13 | SbTCP15 | 58.59 |

**Supplementary Table 2.** GC content at first, second and third codon positions in TCP genes of *Sorghu.*

| S. No. | Gene Model | % GC in 1st codon position | % GC in 2nd codon position | % GC in 3rd codon position |
|---|---|---|---|---|
| 1 | SbTCP1.1 | 0.67 | 0.57 | 0.87 |
| 2 | SbTCP1.2 | 0.67 | 0.57 | 0.87 |
| 3 | SbTCP2 | 0.62 | 0.53 | 0.77 |
| 4 | SbTCP3 | 0.7 | 0.57 | 0.88 |
| 5 | SbTCP4 | 0.71 | 0.63 | 0.92 |
| 6 | SbTCP5 | 0.72 | 0.52 | 0.78 |
| 7 | SbTCP6 | 0.77 | 0.58 | 0.82 |
| 8 | SbTCP7.1 | 0.69 | 0.56 | 0.86 |
| 9 | SbTCP7.2 | 0.69 | 0.56 | 0.86 |
| 10 | SbTCP7.3 | 0.73 | 0.55 | 0.81 |
| 11 | SbTCP8 | 0.69 | 0.54 | 0.82 |
| 12 | SbTCP9 | 0.71 | 0.46 | 0.88 |
| 13 | SbTCP10 | 0.76 | 0.52 | 0.89 |
| 14 | SbTCP11 | 0.73 | 0.63 | 0.97 |
| 15 | SbTCP12 | 0.71 | 0.61 | 0.81 |
| 16 | SbTCP13 | 0.71 | 0.66 | 0.94 |
| 17 | SbTCP14.2 | 0.7 | 0.62 | 0.86 |
| 18 | SbTCP14.3 | 0.7 | 0.62 | 0.86 |
| 19 | SbTCP15 | 0.73 | 0.64 | 0.96 |
| 20 | SbTCP16 | 0.71 | 0.56 | 0.83 |
| 21 | SbTCP17 | 0.74 | 0.57 | 0.92 |
| 22 | SbTCP18 | 0.71 | 0.58 | 0.85 |
| 23 | SbTCP19.1 | 0.69 | 0.51 | 0.82 |
| 24 | SbTCP19.2 | 0.69 | 0.51 | 0.82 |
| 25 | SbTCP19.3 | 0.7 | 0.5 | 0.84 |
| 26 | SbTCP19.4 | 0.7 | 0.5 | 0.84 |
| 27 | SbTCP20 | 0.69 | 0.59 | 0.8 |

**Supplementary Table 3.** Predicted subcellular localization of *Sorghum* TCP proteins (High confidence predictions are highlighted in yellow)

| Gene Name | Final Prediction | Plant-mPLoc | WoLFP SORT | WoLFP SORT Confidence (No of Nearest Neighbours) | DISTILL | DISTILL confidence | MultiLoc2 | MultiLoc2 Confidence | SubLoc v1.0 | SubLoc v1.0 Expected accuracy | CELLO v.2.5 | CELLO v.2.5 Reliability |
|---|---|---|---|---|---|---|---|---|---|---|---|---|
| SbTCP1.1 | Nuclear | Nuclear | Nuclear | 12 | Chloroplast | medium | Cytoplasmic | 0.5 | Nuclear | 97% | Nuclear | 3.073 |
| SbTCP1.2 | Nuclear | Nuclear | Nuclear | 12 | Chloroplast | medium | Cytoplasmic | 0.5 | Nuclear | 97% | Nuclear | 3.073 |
| SbTCP2 | Nuclear | Nuclear | Nuclear | 14 | Nuclear | high | Cytoplasmic | 0.43 | Nuclear | 100% | Nuclear | 4.589 |
| SbTCP3 | Nuclear | Nuclear | Nuclear | 14 | Chloroplast | medium | Cytoplasmic | 0.35 | Nuclear | 94% | Nuclear | 3.222 |
| SbTCP4 | Chloroplast | Chloroplast, Nuclear | Chloroplast, Cytosol | 7, 7 | Chloroplast | high | mitochondrial | 0.47 | Extracellular | 91% | Nuclear | 2.035 |
| SbTCP5 | Nuclear | Nuclear | Nuclear | 13 | Nuclear | medium | Cytoplasmic | 0.56 | Nuclear | 91% | Nuclear | 3.307 |
| SbTCP6 | - | Nuclear | Nuclear | 7 | Chloroplast | medium | Cytoplasmic | 0.49 | Extracellular | 91% | Nuclear | 3.331 |
| SbTCP7.1 | Nuclear | Nuclear | Nuclear | 14 | Nuclear | high | Chloroplast | 0.48 | Nuclear | 91% | Nuclear | 4.137 |
| SbTCP7.2 | Nuclear | Nuclear | Nuclear | 14 | Nuclear | high | Chloroplast | 0.48 | Nuclear | 91% | Nuclear | 4.137 |
| SbTCP7.3 | Nuclear | Nuclear | Chloroplast | 10 | Chloroplast | medium | Secretory pathway | 0.4 | Nuclear | 95% | Nuclear | 4.299 |
| SbTCP8 | Nuclear | Nuclear | Nuclear | 12.5 | Nuclear | medium | Secretory pathway | 0.51 | Nuclear | 84% | Nuclear | 4.274 |
| SbTCP9 | - | Nuclear | Nuclear | 7 | Chloroplast | medium | Secretory pathway | 0.48 | Nuclear | 84% | Nuclear | 3.515 |
| SbTCP10 | Cytoplasmic | Nuclear | Nuclear | 6 | Chloroplast | medium | Cytoplasmic | 0.78 | Nuclear | 94% | Nuclear | 3.412 |
| SbTCP11 | - | Nuclear | Nuclear | 7 | Chloroplast | medium | Chloroplast | 0.37 | Mitochondrial | 84% | Nuclear | 2.09 |
| SbTCP12 | Nuclear | Nuclear | Nuclear | 14 | Nuclear | medium | Cytoplasmic | 0.53 | Nuclear | 94% | Nuclear | 4.161 |
| SbTCP13 | - | Nuclear | Nuclear | 10.5 | Chloroplast | medium | Chloroplast | 0.45 | Extracellular | 84% | Nuclear | 2.379 |
| SbTCP14.2 | Chloroplast | Nuclear | Chloroplast | 9 | Chloroplast | medium | Chloroplast | 0.71 | Nuclear | 74% | Nuclear, Chloroplast | 1.702, 1.352 |
| SbTCP14.3 | - | Nuclear | Chloroplast | 9 | Chloroplast | medium | Chloroplast | 0.41 | Nuclear | 74% | Nuclear, Chloroplast | 1.702, 1.352 |
| SbTCP15 | Nuclear/chloroplast | Nuclear | Nuclear | 7 | Chloroplast | low | Chloroplast | 0.81 | Nuclear | 100% | Nuclear | 3.46 |
| SbTCP16 | Nuclear | Nuclear | Nuclear | 9 | Nuclear | high | Cytoplasmic | 0.49 | Mitochondrial | 94% | Nuclear | 1.698 |
| SbTCP17 | Nuclear | Nuclear | Nuclear | 13 | Chloroplast | medium | Cytoplasmic | 0.36 | Extracellular | 91% | Nuclear | 2.905 |
| SbTCP18 | - | Nuclear | Nuclear | 11 | Nuclear | medium | Chloroplast | 0.41 | Extracellular | 56% | Nuclear | 2.724 |
| SbTCP19.1 | - | Nuclear | Nuclear, cytosol | 5, 5 | Chloroplast | medium | Chloroplast | 0.38 | Nuclear | 94% | Nuclear, Mitochondrial | 1.779, 1.340 |
| SbTCP19.2 | - | Nuclear | Nuclear, cytosol | 5, 5 | Chloroplast | medium | Chloroplast | 0.38 | Nuclear | 94% | Nuclear, Mitochondrial | 1.779, 1.340 |

| | | | | | | | | | | | | |
|---|---|---|---|---|---|---|---|---|---|---|---|---|
| SbTCP19.3 | - | Nuclear | Nuclear, cytosol | 5, 5 | Chloroplast | low | Chloroplast | 0.41 | Nuclear | 91% | Nuclear, Mitochondrial | 1.726, 1.388 |
| SbTCP19.4 | - | Nuclear | Cytosol | 5 | Chloroplast | low | Chloroplast | 0.41 | Nuclear | 91% | Nuclear, Mitochondrial | 1.726, 1.388 |
| SbTCP20 | Nuclear | Cell membrane, Nuclear | Nuclear | 14 | Nuclear | medium | Cytoplasmic | 0.54 | Nuclear | 91% | Nuclear | 4.322 |

**Supplementary Table 4.** Results of Orthology analysis of *Sorghum* proteins with rice and *Arabidopsis*.

| Sorghum Gene | Locus ID | Rice Gene Name | % Similarity | E-value | Score |
|---|---|---|---|---|---|
| SbTCP1 | LOC_Os03g57190 | PCF6 | 60.05 | 1E-108 | 327 |
| | LOC_Os07g05720 | OsTCP21 | 45.79 | 2E-70 | 231 |
| SbTCP2 | LOC_Os03g49880 | OsTB1(Lateral Branching 1) | 57.11 | 2E-116 | 348 |
| SbTCP3 | LOC_Os07g05720 | OsTCP21 | 61.57 | 1E-118 | 358 |
| | LOC_Os03g57190 | PCF6 | 46.3 | 8E-69 | 227 |
| SbTCP4 | NA | NA | NA | NA | NA |
| SbTCP5 | LOC_Os09g24480 | OsTCP24 | 61.86 | 5E-61 | 197 |
| | LOC_Os08g33530 | OsTCP22 | 59.84 | 3E-31 | 119 |
| SbTCP6 | LOC_Os08g43160 | PCF2 | 62.04 | 2E-77 | 245 |
| | LOC_Os09g34950 | OsTCP25 | 56.91 | 3E-75 | 239 |
| SbTCP7 | LOC_Os01g11550 | PCF5 | 76.73 | 2E-178 | 512 |
| SbTCP8 | LOC_Os01g55100 | PCF7 | 62.89 | 6E-72 | 232 |
| | LOC_Os01g11550 | PCF5 | 36.8 | 5E-39 | 148 |
| SbTCP9 | LOC_Os01g55750 | OsTCP5 | 66.67 | 2E-100 | 300 |
| | LOC_Os05g43760 | OsTCP18 | 47.86 | 2E-58 | 192 |
| SbTCP10 | LOC_Os01g69980 | OsTCP6 | 67.59 | 4E-93 | 282 |
| | LOC_Os02g51280 | OsTCP9 | 87.14 | 1E-36 | 138 |
| | LOC_Os06g12230 | OsTCP19 | 85.51 | 4E-36 | 136 |
| | LOC_Os11g07460 | PCF3 | 74.44 | 9E-35 | 133 |
| | LOC_Os12g07480 | OsTCP28 | 66.67 | 7E-34 | 130 |
| SbTCP11 | LOC_Os02g42380 | OsTCP7 | 68.2 | 5E-69 | 214 |

| | | | | | |
|---|---|---|---|---|---|
| | LOC_Os04g44440 | OsTCP17 | 56.64 | 3E-58 | 186 |
| SbTCP12 | LOC_Os02g51280 | OsTCP9 | 86.13 | 8E-162 | 465 |
| | LOC_Os06g12230 | OsTCP19 | 64.85 | 9E-105 | 318 |
| | LOC_Os01g69980 | OsTCP6 | 69.7 | 1E-38 | 142 |
| SbTCP13 | LOC_Os04g44440 | OsTCP17 | 45.32 | 3E-36 | 130 |
| | LOC_Os12g07480 | OsTCP28 | 85.71 | 7E-36 | 133 |
| | LOC_Os11g07460 | PCF3 | 73.56 | 2E-35 | 132 |
| | LOC_Os02g42380 | OsTCP7 | 90.48 | 1E-34 | 126 |
| SbTCP14 | LOC_Os04g11830 | PCF1 | 60.64 | 1E-52 | 169 |
| | LOC_Os11g07460 | PCF3 | 65.38 | 1E-28 | 111 |
| SbTCP15 | LOC_Os04g44440 | OsTCP17 | 67.89 | 1E-75 | 230 |
| | LOC_Os02g42380 | OsTCP7 | 55.11 | 1E-53 | 174 |
| | LOC_Os11g07460 | PCF3 | 53.39 | 4E-30 | 116 |
| SbTCP16 | LOC_Os08g33530 | OsTCP22 | 46.64 | 3E-46 | 158 |
| | LOC_Os09g24480 | OsTCP24 | 88.73 | 5E-35 | 129 |
| SbTCP17 | LOC_Os08g43160 | PCF2 | 75.46 | 2E-156 | 449 |
| | LOC_Os09g34950 | OsTCP25 | 59.93 | 4E-74 | 238 |
| | LOC_Os11g07460 | PCF3 | 73.49 | 1E-32 | 127 |
| SbTCP18 | LOC_Os12g42190 | PCF8 | 64.42 | 2E-87 | 267 |
| | LOC_Os12g02090 | OsTCP27 | 80.46 | 1E-36 | 131 |
| | LOC_Os07g05720 | OsTCP21 | 57.46 | 1E-31 | 124 |
| | LOC_Os03g57190 | PCF6 | 73.17 | 7E-31 | 120 |
| SbTCP19 | LOC_Os05g43760 | OsTCP18 | 61.61 | 6E-94 | 284 |
| | LOC_Os01g55750 | OsTCP5 | 51.37 | 1E-66 | 214 |
| SbTCP20 | LOC_Os02g51280 | OsTCP9 | 68.3 | 1E-124 | 370 |
| | LOC_Os06g12230 | OsTCP19 | 75.83 | 5E-117 | 350 |
| | LOC_Os12g07480 | OsTCP28 | 91.03 | 8E-41 | 151 |
| | LOC_Os11g07460 | PCF3 | 85.37 | 1E-40 | 152 |

| | | | | | |
|---|---|---|---|---|---|
| | LOC_Os01g69980 | OsTCP6 | 75.58 | 2E-37 | 139 |

| Sorghum Gene | Gene ID | Arabidopsis Gene | % Similarity | E-value | Score |
|---|---|---|---|---|---|
| SbTCP1 | AT4G18390 | AtTCP2 | 42.12 | 2E-40 | 149 |
| | AT1G30210 | AtTCP24 | 41.08 | 2E-38 | 142 |
| | AT3G02150 | AtTCP13 | 65.85 | 4E-28 | 112 |
| SbTCP2 | AT3G18550 | AtTCP18 | 38.05 | 2E-25 | 106 |
| SbTCP3 | AT4G18390 | AtTCP2 | 58.58 | 5E-49 | 174 |
| | AT1G30210 | AtTCP24 | 50.78 | 2E-42 | 155 |
| SbTCP4 | NA | NA | NA | NA | NA |
| SbTCP5 | AT3G18550 | AtTCP18 | 63.1 | 6E-28 | 111 |
| SbTCP6 | AT2G45680 | AtTCP9 | 40.16 | 1E-38 | 142 |
| SbTCP7 | AT3G15030 | AtTCP4 | 41.86 | 9E-75 | 244 |
| | AT1G53230 | AtTCP3 | 40.19 | 2E-68 | 226 |
| | AT2G31070 | AtTCP10 | 47.39 | 2E-43 | 159 |
| SbTCP8 | AT3G15030 | AtTCP4 | 40.73 | 2E-39 | 148 |
| | AT1G53230 | AtTCP3 | 36.8 | 3E-39 | 147 |
| | AT2G31070 | AtTCP10 | 79.71 | 3E-30 | 120 |
| SbTCP9 | AT5G60970 | AtTCP5 | 81.48 | 2E-39 | 143 |
| | AT3G02150 | AtTCP13 | 58.33 | 2E-38 | 139 |
| | AT5G08070 | AtTCP17 | 63.27 | 1E-37 | 136 |
| SbTCP10 | AT3G27010 | AtTCP20 | 50.84 | 5E-53 | 179 |
| | AT1G58100 | AtTCP8 | 71.28 | 9E-37 | 138 |
| | AT1G69690 | AtTCP15 | 85.51 | 1E-36 | 136 |
| | AT2G45680 | AtTCP9 | 76.83 | 6E-36 | 135 |
| | AT3G47620 | AtTCP14 | 86.76 | 8E-36 | 136 |
| SbTCP11 | AT5G23280 | AtTCP7 | 44.59 | 2E-40 | 141 |
| | AT5G08330 | AtTCP21 | 42.03 | 1E-35 | 128 |
| SbTCP12 | AT3G47620 | AtTCP14 | 43.47 | 8E-58 | 199 |
| | AT1G69690 | AtTCP15 | 68.5 | 5E-52 | 179 |
| | AT1G58100 | AtTCP8 | 85.88 | 6E-45 | 162 |
| | AT1G72010 | AtTCP22 | 39.81 | 1E-42 | 155 |
| SbTCP13 | AT5G23280 | AtTCP7 | 63.13 | 5E-77 | 236 |
| | AT1G35560 | AtTCP23 | 77.78 | 3E-37 | 136 |
| SbTCP14 | NA | NA | NA | NA | NA |
| SbTCP15 | AT5G23280 | AtTCP7 | 45.81 | 9E-39 | 136 |
| | AT5G08330 | AtTCP21 | 80.82 | 2E-35 | 127 |
| SbTCP16 | AT3G18550 | AtTCP18 | 49.18 | 5E-28 | 111 |
| SbTCP17 | AT2G45680 | AtTCP9 | 44.54 | 5E-47 | 166 |
| | AT5G51910 | AtTCP19 | 44.66 | 2E-42 | 152 |
| | AT1G72010 | AtTCP22 | 71.43 | 1E-34 | 132 |
| SbTCP18 | AT4G18390 | AtTCP2 | 51.85 | 4E-30 | 118 |
| SbTCP19 | AT5G60970 | AtTCP5 | 86.67 | 1E-39 | 144 |
| | AT3G02150 | AtTCP13 | 52.41 | 1E-39 | 142 |
| | AT5G08070 | AtTCP17 | 80.26 | 4E-37 | 134 |
| SbTCP20 | AT3G47620 | AtTCP14 | 41.52 | 7E-56 | 193 |
| | AT1G69690 | AtTCP15 | 67.97 | 3E-49 | 172 |
| | AT1G58100 | AtTCP8 | 84.71 | 2E-44 | 160 |
| | AT1G35560 | AtTCP23 | 86.05 | 2E-42 | 154 |
| | AT1G72010 | AtTCP22 | 85.19 | 3E-41 | 151 |

**Supplementary Figure 1.** Results of MEME motif analysis with TCP proteins from *Arabidopsis*, rice, *Brachypodium* and *Sorghum*.

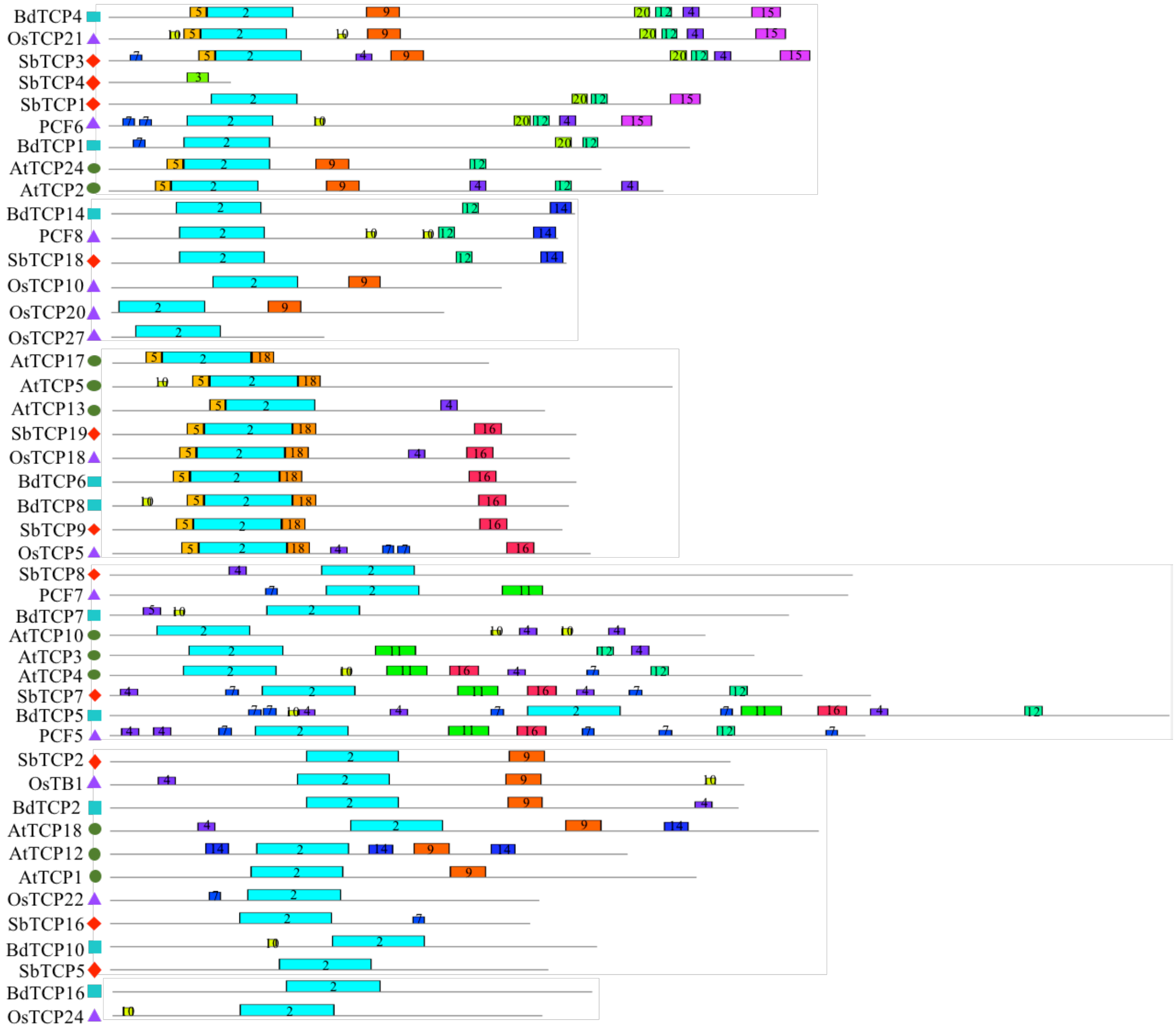

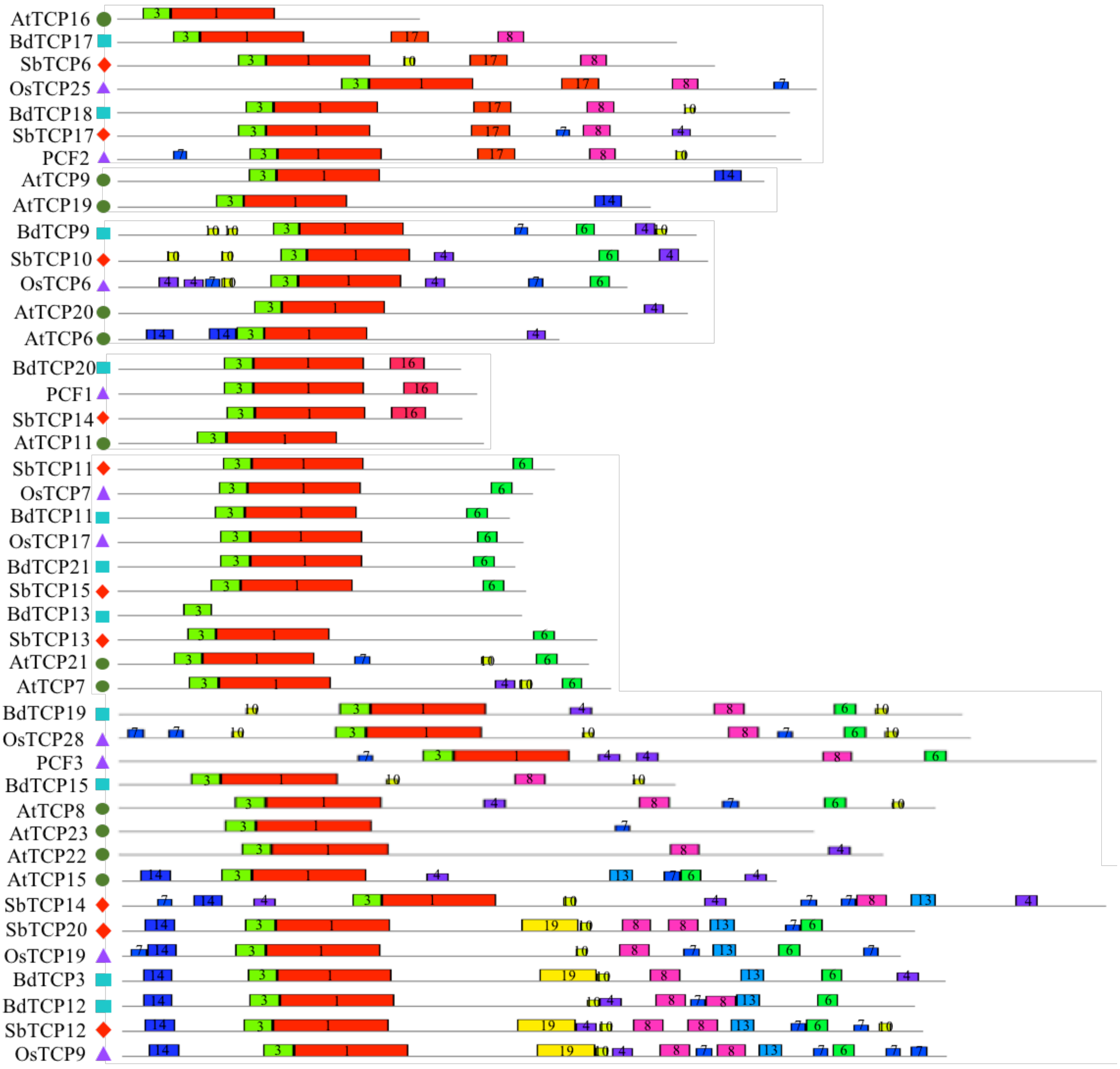
AtTCP16
BdTCP17
SbTCP6
OsTCP25
BdTCP18
SbTCP17
PCF2
AtTCP9
AtTCP19
BdTCP9
SbTCP10
OsTCP6
AtTCP20
AtTCP6
BdTCP20
PCF1
SbTCP14
AtTCP11
SbTCP11
OsTCP7
BdTCP11
OsTCP17
BdTCP21
SbTCP15
BdTCP13
SbTCP13
AtTCP21
AtTCP7
BdTCP19
OsTCP28
PCF3
BdTCP15
AtTCP8
AtTCP23
AtTCP22
AtTCP15
SbTCP14
SbTCP20
OsTCP19
BdTCP3
BdTCP12
SbTCP12
OsTCP9

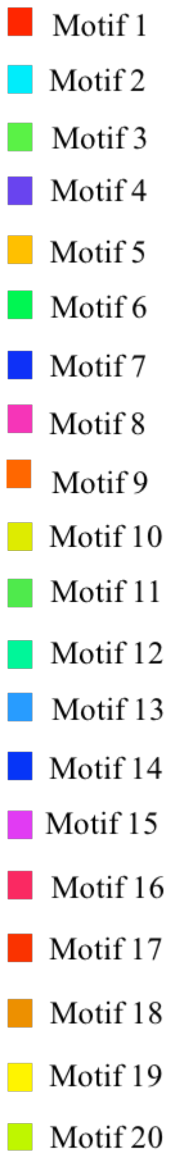
Motif 1
Motif 2
Motif 3
Motif 4
Motif 5
Motif 6
Motif 7
Motif 8
Motif 9
Motif 10
Motif 11
Motif 12
Motif 13
Motif 14
Motif 15
Motif 16
Motif 17
Motif 18
Motif 19
Motif 20
